# Eye-Opening Effect Achieved by Modified Transconjunctival Lower Blepharoplasty

**DOI:** 10.1093/asj/sjae205

**Published:** 2024-10-17

**Authors:** Takayuki Kubo

## Abstract

**Background:**

Enophthalmia (abnormally sunken eyeball in the socket) and ptotic upper lid, as well as lower lid symptoms, are commonly observed in patients seeking periorbital rejuvenation.

**Objectives:**

The aim of this study was to assess the effect of transconjunctival lower blepharoplasty (TCLB) modified by adding deframing and decompression maneuvers to the lower orbital fat compartment (LOFC) and its support structures to obtain better results in both the lower and upper lids.

**Methods:**

Modified TCLB was performed in patients with lower eyelid symptoms. Palpebral fissure height (PFH) (the distance between the upper and lower eyelids in vertical alignment with the center of the pupil) was measured before surgery and 12 months postoperatively from 3-dimensional photographs. These data were compared to validate the postoperative eye-opening effect. The weight of the excised fat from each LOFC was also measured and compared.

**Results:**

Forty patients (36 females and 4 males) who underwent modified TCLB were followed up 12 months postoperatively. Mean [standard deviation] preoperative PFHs were 8.41 [1.15] mm (range, 6.1-10.7 mm) for the right and 8.41 [1.12] mm (range, 5.5-10.4 mm) for the left. Postoperative PFHs were 9.26 [0.95] mm (range, 6.4-11.1 mm) for the right and 9.21 [0.94] mm (range, 6.2-11.1 mm) for the left. The improvement in postoperative eye opening was statistically significant. The total excised LOFC was 0.43 [0.24] g (range, 0-1.2 g) for the right and 0.42 [0.25] g (range, 0-1.5 g) for the left. The largest amount of fat was excised bilaterally from the lateral LOFC, and the difference was statistically significant.

**Conclusions:**

The results after the modified TCLB clearly demonstrate increased eye-opening ability and marked resolution of observable symptoms. The anatomical dynamics of the orbit involved in this procedure are detailed through scientific data.

**Level of Evidence: 4 (Therapeutic):**

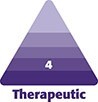

Signs of aging around the eye initially appear in the periorbital regions due to the intricate anatomical structures there.^1^ For instance, the upper eyelid blinks almost 20,000 times a day, and these repeated mechanical actions wear skin from the upper eyelids with age because the thin periorbital skin is easily attenuated, leading to the manifestation of droopy upper eyelids or wrinkles as evidence of premature aging. Symptoms also appear on the lower eyelids and typically manifest as baggy eyelids (anterior herniation of the lower orbital fat compartment [LOFC]), tear trough deformities, nasojugal grooves, and dark circles.^2-4^ Tear trough deformity is a hollowed and sunken area between the lower lid and cheekbone that originates in the inner canthus and extends to the midpupillary line. Depression lateral to tear trough deformity is also referred to as the nasojugal groove. Dark circles are unique to the lower lid and show inverted semilunar-shaped dark shadows that delineate the lower orbit. This is further highlighted by the combination of skin pigmentations. Dark circles are occasionally exaggerated by other lower lid symptoms, such as eyebags and tear trough deformities. Through meticulous studies of the periorbital region, the anatomical structures and functions of the eyelid have been thoroughly investigated and documented.^5-7^ Treating aging signs in lower lids has traditionally been performed by cutting-edge surgeries, making oculoplastic surgery one of the most common procedures performed by facial aesthetic surgeries.^8-10^ Transconjunctival lower blepharoplasty (TCLB) is becoming a high-profile surgery due to its minimal invasiveness.^11^ In particular, scar-free surgery is favored in Asian populations, because this skin type is more susceptible to postoperative scarring.^12,13^

TCLB can offer a straightforward solution to remedy periorbital problems when symptoms of the upper and lower eyelids are considered separately. However, if complex symptoms are present that involve both the upper and lower lids, the solution is more complex. For instance, enophthalmia is an abnormality of the sunken globe in the orbital socket caused by age-related attenuation of the supporting tissues inside the lower orbit.^14^ It shows not only a typical aging appearance but is also believed to cause pseudoblepharoptosis or dermatochalasis. Although several studies have been conducted on enophthalmia since the late 1990s, its pathophysiology and treatment remain controversial. As the number of periorbital surgeries increased in the author’s daily practice, there was a growing number of patients with concomitant periorbital symptoms such as sunken globe, dermatochalasis, ptotic upper lids, and lower lid problems. In our practice, patients who had symptoms in both eyelids were primarily treated with a modified TCLB rather than upper lid surgery, achieving improvements in symptoms of both lids with an increased eye-opening effect. In this article, indirect improvement in upper lid symptoms by performing a modified TCLB is scientifically proven, and the anatomical dynamics underlying this phenomenon are discussed.

## METHODS

This study followed the Declaration of Helsinki and its guiding principles. Modified TCLB was performed in patients who visited our clinic for the treatment of lower eyelid symptoms between January 2022 and December 2022. This study received IRB approval authorized by the Japan Registry of Clinical Trials at the Ministry of Health, Labour and Welfare of Japan (CRB1180001).

Preoperative examination was performed in the seated position and candidates were classified into either “eyebags,” “tear trough deformity” or “nasojugal groove,” and “dark circles” of the lower eyelid. The upper eyelids were also examined for enophthalmia, drooping of the upper lid (dermatochalasis), or blepharoptosis. Currently, there are no definitive diagnostic criteria for enophthalmia. If the preoperative eye protrusion value measured by the Hertel exophthalmometer was less than 10 mm, the patient was tentatively classified as enophthalmic because the mean protrusion value is 14.4 mm (range, 8-20 mm) in Asians.^15,16^ In the case of blepharoptosis, the condition was precisely evaluated, and patients with severe blepharoptosis (margin-reflex distance 1 < −0.5 mm) were excluded from this study. Margin-reflex distance 1 is the distance from the upper eyelid margin to the corneal light reflex in the primary position, and its normal range is 2.2-5.5 mm. Patients with unilateral blepharoptosis were excluded from the study. Patients who had undergone previous upper lid surgery or subsequent upper blepharoplasty during the 12-month follow-up period were also excluded. Forty patients who fulfilled the above criteria and could attend follow-up appointments up to 12 months postoperatively were included in the study.

**Figure 1.**
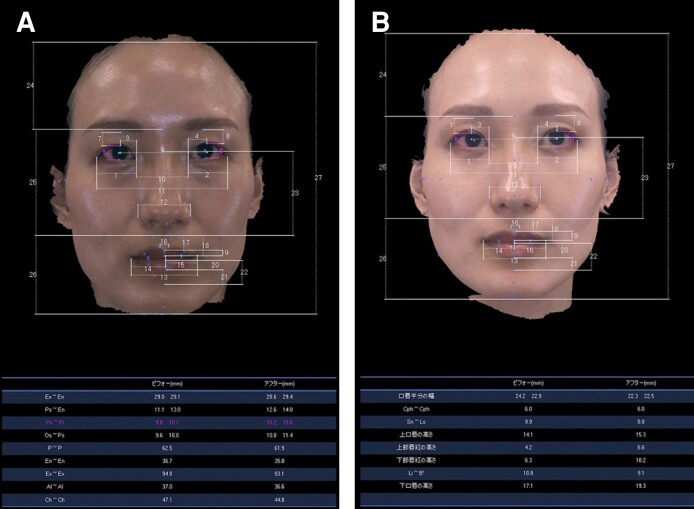
Preoperative and 12-month postoperative palpebral fissure heights measured by 3-dimensional photographic analysis. The patient is a 34-year-old female who showed improvement in eye opening 12 months postoperatively from 9.8 mm to 10.2 mm on the right (A) 10.1 mm to 10.6 mm on the left (B). Ex, exocanthion; En, endocanthion; Ps, pulpebrale superius; Pi, pulpebrale inferius; Os, orbitale superius; P, pupil; AI, alare; Ch, cheilion; Cph, critsta philtri; Sn, subnasale; Ls, labiale superius; Li, labiale Inferius; B', Sublabiale.

One surgeon performed the entire procedure, including 12 months of postoperative follow-up. All candidates had a northern Asian background. The contraindication for this surgery was a predisposition to severe bleeding. For patients prescribed with antithrombotic drugs, intake was withdrawn 5 days prior to surgery. Informed consent was obtained, and all associated risks were explained prior to surgery. Both plain and 3-dimensional (3D) photographs (captured with an imaging system produced by Morpheus Co. Ltd., Yongin, Gyeonggi, South Korea) were obtained preoperatively and at each postoperative visit. 3D photographs were taken in a controlled environment with the patient sitting in a fixed position in the room, with matched lighting, distance, and seating height. While the photographs were being taken, patients were instructed not to elevate the eyebrow to assist in opening the eye to ensure fair assessment of the surgical effect on the periorbital regions. Twelve-month postoperative 3D photographs were compared with preoperative photographs to identify eye-opening effects, and evaluation of the outcome variables were performed once, at 12 months postsurgery (Figure 1).

Improvement in postoperative lower lid symptoms was not objectively evaluated in this study. Instead, this was subjectively assessed using a satisfaction rate test. The survey used in this study was conducted on paper and was anonymous. The survey was distributed by a medical assistant working at the author’s clinic. All patients were asked to rate their satisfaction with the results at the 12-month postoperative follow-up appointment. The scale ranged from 0 to 3 (0 = poor, 1 = no difference, 2 = better, 3 = much better). The survey sheets are available as Supplemental Files, Appendix A and Appendix B, respectively.

### Statistical Analysis

Preoperative and postoperative palpebral fissure heights (PFHs) were measured and statistically compared by means of a paired *t*-test. These measurements and analyses were performed by the author using Morpheus software with the help of a technician at Morpheus Co. Ltd. Fat pads excised from the bilateral (nasal, central, and lateral) parts of the LOFC of 40 patients during the modified TCLB were collected and weighed. Differences in excised fat weight from each LOFC were statistically compared using the Friedman test and a Wilcoxon signed-rank sum post-hoc test.

### Surgical Technique

Before commencing surgery, the lower lid was evaluated to verify the presence of eyebags, tear trough deformity or nasojugal groove, and dark circles. The surgical design for approximate LOFC positions was developed by using a marker pen on the lower lid surface. After establishing the intravenous line, the patient was placed in the supine position. Mild sedation with diazepam (10-20 mg) and hydroxyzine hydrochloride (12.5-25 mg) was administered to relax the patient. Throughout the surgery, blood pressure and other vital signs were closely monitored. After disinfecting and draping the face, approximately 6 mL of 1% xylocaine with 1:100,000 epinephrine was injected into each lower orbital compartment through the conjunctiva while the eye was closed, ensuring that the lower orbit was fully bulged to allow for easy surgical maneuvering. The lower eyelid was flipped, and stay sutures were placed at the lower margin of the tarsus and inferior conjunctival fornix. Both stay sutures were pulled with vertical traction to expose the entire conjunctiva of the lower lid for full surgical manipulation. The overall surgical procedure for the modified TCLB is shown in Video 1.

**Figure 2.**
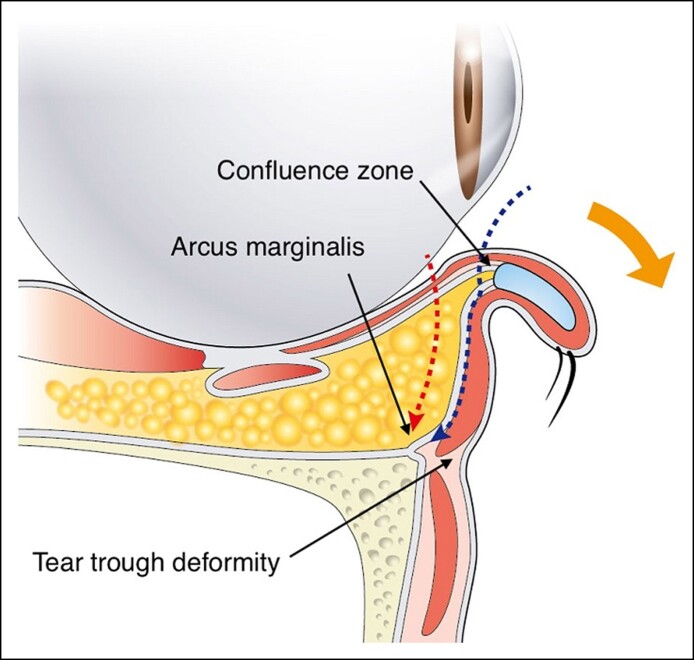
Illustration of sagittal plane of the inverted lower eyelid. The dotted line over the septum is the preseptal approach entering from the confluence zone of the capsulopalpebral fascia and orbital septum 5 mm inferior to the tarsus. Dissection was performed inferiorly under the preseptal orbicularis oculi muscle until reaching the edge of the inferior orbital rim over the arcus marginalis and below the tear trough deformity. The dotted line inside the LOFC represents the postseptal approach transecting the conjunctiva approximately 6 mm inferior to the tarsus, directly reaching the center of the LOFC.

**Figure 3.**
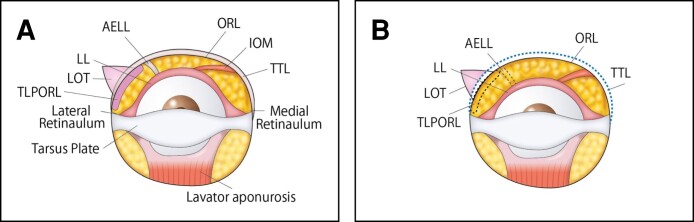
Schematic view of the deframing process during modified TCLB (the left orbital socket is shown upside-down). (A) Preoperative illustration. Underlying lower lid structures are shown after dissection of the orbital septum. When dissection of the preseptal space reached the inferior orbital rim, the AELL is identified between the central and lateral LOFC inside the lower orbital socket and inserted into the posterior lateral orbital socket, while the IOM running between the nasal and central LOFCs is observed. The TLPORLs also merge into the AELL and LL back and downward to the lateral retinaculum. The entire LOFC is still framed and squeezed into a narrow space surrounded by supporting tissues inside the orbital socket. (B) Postoperative illustration. Deframing and decompression procedures are performed inside the lower orbit. The tear trough ligament (TTL) at the nasal part of the inferior orbital rim and the ORL at the central and lateral parts are fully released. The AELL is transected, the TLPORL is dissected from the confluence point of the lateral retinaculum to the AELL, and the proximal edge of the LOT over the orbital rim is slightly dissected. Due to these deframing maneuvers for the LOFC, the central and lateral LOFCs gain mobility as a single composite, and all LOFCs become more manageable. Dissected and transected ligaments are indicated as dotted lines. AELL, arcuate expansion of Lockwood's ligament; LOFC, lower orbital fat compartment; IOM, inferior oblique muscle; LL, Lockwood's ligament; LOT, lateral orbital thickening; ORL, orbicularis retaining ligament; TCLB, transconjunctival lower blepharoplasty; TLPORL, thicker lateral part of orbicularis retaining ligament.

A horizontal transection was made on the conjunctiva within 5 mm of the tarsus using a radiofrequency electrode probe in the direction of the fusion zone between the capsulopalpebral fascia and orbital septum by the preseptal approach, as shown in Figure 2. The dissection continued through the layer between the orbital septum and the orbital part of the orbicularis oculi until the confluence point of the orbicularis retaining ligament (ORL) (the tear trough ligament in the nasal region) and the arcus marginalis at the edge of the inferior orbital rim. The tear trough ligament and ORL were released medially and laterally over the lower orbital rim to allow full exposure of the orbital septum holding the LOFC. The orbital septum was transected to reach the LOFC. Arcuate expansion of Lockwood's ligament (AELL) was identified directly under the septum, running between the central and lateral LOFCs. The AELL was also released and transected to reduce the tension between the central and lateral LOFCs. As a result, increased mobility of the central and lateral LOFCs was achieved as a single unit. Deep dissection was continued posterolaterally over the lateral LOFC until the bone surface of the lateral orbital socket. During dissection, identification was confirmed at the AELL merging with the Lockwood's ligament (LL) and the ORL posteroinferior to the lateral canthal tendon (retinaculum). The thicker lateral part of the ORL (TLPORL) and the proximal edge of the lateral orbital thickening (LOT) over the orbital rim were slightly dissected to facilitate mobility and redraping of the superficial orbicularis fascia, and the lateral retinaculum was partially transected to decompress the lateral LOFC. Dissection and transection facilitated LOFC mobility, releasing the lateral LOFC squeezed into the restricted space surrounding the TLPORL, AELL, and LL in the posterolateral orbital socket.^17^ The anatomical structures involved in these procedures are shown in Figure 3. Excess parts of the central and lateral LOFCs were resected until the surface was level with the inferior orbital rim. The critical procedures performed in the lateral lower orbit are shown in Video 2.

The nasal LOFC is located medially to the inferior oblique muscle and is easily identified by its pale coloration compared with other LOFCs. If the nasal LOFC was prolific, the distal part was resected to reduce the inner bulging of the lower lid. After decompression and resection of each LOFC, transpositioning and redraping of the nasal and central LOFC over the inferior orbital rim were performed in cases where the patient had a distinct tear trough deformity. In these patients, if sufficient extirpated LOFC fat was present, it was minced as free microfat pellets for transplantation into the nasal and central infraorbital rims to fill the gap. In cases where the nasal LOFC was thick and tall, a pedicle flap was raised, rotated, and placed down to the infraorbital rim. With these manipulations, the flatness of the anterior surface of the LOFC was confirmed. Manipulation of the nasal LOFC is shown at the end of Video 1.

Careful hemostasis was performed during surgery to maintain a bloodless surgical field. A ball-shaped electrode probe was applied to the surface of the orbital socket in instances of bone bleeding. If complete hemostasis was not possible, bone wax (Ethicon Inc., NJ) was applied to ensure reliable hemostasis. Antibiotic eyedrops were administered to the surgical area at the conclusion of the surgery. Stay sutures were released to flip the lower lid back to its original position. The surgery was completed without placement of conjunctival sutures. The patient was placed in a reclining supine position, and the eyes were compressed with ice packs for at least 1 hour postoperatively. Complete hemostasis was reconfirmed, and immediate postoperative photographs were obtained before the patient was discharged.

## RESULTS

The modified TCLB was performed by the author in 300 patients between January 2022 and December 2022. The mean [standard deviation] age of the patients was 46.94 [11.86] years (range, 18-78 years); 241 were female, aged 46.29 [11.8] years (range, 18-78 years) and 59 were male, aged 49.59 [11.81] years (range, 20-75 years). Forty patients (36 females and 4 males) were followed up for 12 months postoperatively (Table 1). The number of lower lid symptoms and other concurrent orbital symptoms were recorded. Eyebags in the lower lid were the most common (35%), followed by a combination of dark circles (10%) or tear trough deformity (nasojugal fold) (10%) with eyebags, as shown in Table 2. Enophthalmia or its tendency was the most common concurrent orbital symptom (30%), followed by no symptoms (22.5%), and enophthalmia with either dermatochalasis (12.5%) or blepharoptosis (12.5%) (Table 3). Of the 42 followed-up patients, the average surgery time was 19 minutes 23 seconds [3 minutes 57 seconds] (range, 14 minutes 7 seconds-28 minutes 19 seconds) on the right side and 20 minutes 1 second [3 minutes 54 seconds] (range, 14 minutes 36 seconds-27 minutes 20 seconds) on the left side. Patients were followed up and tracked postoperatively at intervals of 1, 3, 6, and 12 months. Follow-up was concluded 12 months after the initial appointment. Two patients were excluded from the study: 1 patient had a previous upper blepharoplasty within 3 months before the surgery, and the other was an elderly patient who during the follow-up period had a subsequent transcutaneous lower blepharoplasty for excess skin management.

**Table 1.** Patient Demographics

	Female	Male	Total
Number of patients	241 (80.3%)	59 (19.6%)	300
Mean age (years)	46.29 [11.8] (18-78)	49.59 [11.81] (20-75)	46.94 [11.86] (18-78)
Number of follow-up patients	36 (90%)	4 (10%)	40
Mean age (years)	47.08 [12.87] (26-79)	55 [5.66] (47-59)	47.88 [12.52] (26-79)

Values are n (%) or mean [standard deviation] (range).

**Table 2.** Lower Eyelid Symptoms

	A	A + B	A + D	B + C	A + B + C	A + B + C + D	A + C + D	B + D	C + D	C	Total
Number of patients	14	4	4	3	2	1	1	1	1	1	40

A, eyebags; B, dark circles; C, tear trough and nasojugal fold; D, wrinkles.

**Table 3.** Other Orbital Symptoms

	A	N	A + B	A + C	B	D	C	C + D	B + C	Total
Number of patients	13	10	5	5	2	2	1	1	1	40

A, enophthalmia; B, dermatochalasis; C, blepharoptosis; D, exophthalmos; N, no symptoms.

**Figure 4.**
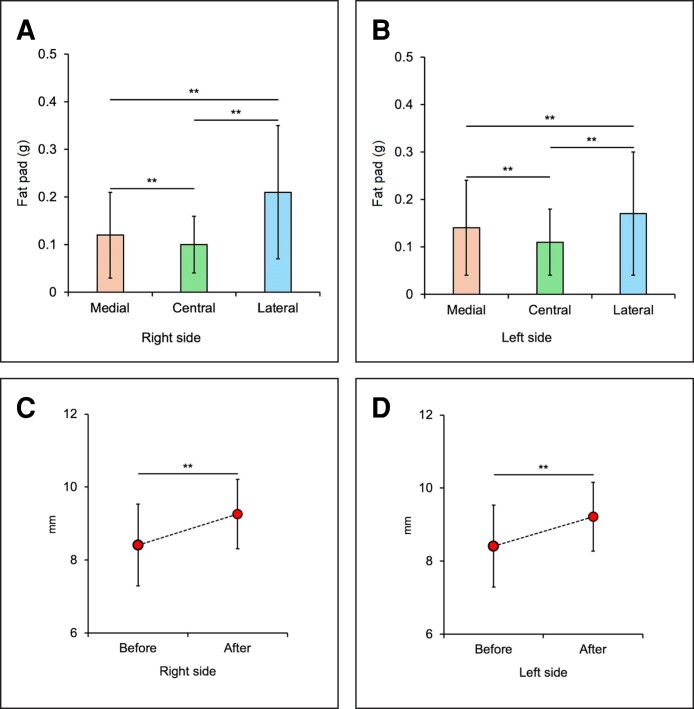
Excised fat pad weight from each LOFC in 40 followed-up patients was compared for right (A) and left (B) sides. Excised fat from the lateral LOFC was the most bilateral. The sides were compared by the Friedman test and a Wilcoxon signed-rank post-hoc test. The excised fat pad weight difference for each LOFC was statistically significant for both sides. Pre- and postoperative PFHs in 40 patients were compared for the left (C) and right (D) sides. The postoperative PFHs were greater on both sides. PFHs were statistically compared by paired *t*-test. **P < .01. LOFC, lower orbital fat compartment; PFH, palpebral fissure height.

The average weights of excised fat from the respective LOFC of 131 patients during the modified TCLB were 0.12 [0.09] g (range, 0-0.4 g) for nasal, 0.1 [0.06] g (range, 0-0.3 g) for central, 0.21 [0.14] g (range, 0-0.6 g) for lateral, and 0.43 [0.24] g (range, 0-1.2 g) in total for the right side and 0.14 [0.1] g (range, 0-0.6 g) for nasal, 0.11 [0.07] g (range, 0-0.4 g) for central, 0.17 [0.13] g (range, 0-0.7 g) for lateral, and 0.42 [0.25] g (range, 0-1.5 g) in total for the left side. These values were compared, showing that fat excised from the lateral LOFC was greater than that excised from other parts of the LOFC on both sides, and the fat excised from the nasal was greater than that excised from the central LOFC. These results are statistically significant (Supplemental Table 1; Figure 4A) The preoperative average PFH was 8.41 [1.15] mm (range, 6.1-10.7 mm) for the right and 8.41 [1.12] mm (range, 5.5-10.4 mm) for the left. Twelve-month postoperative PFHs were 9.26 [0.95] mm (range, 6.4-11.1 mm|) for the right and 9.21 [0.94] mm (range, 6.2-11.1 mm) for the left. The results show that at 12 months postoperatively, the bilateral PFH is significantly greater than the preoperative value (Supplemental Table 2; Figure 4B)

Among the ancillary treatments performed on the lower eyelid in the 300 modified TCLB cases, hyaluronic acid injection into the tear trough or nasojugal groove was most frequently performed (41 cases), followed by Botox injected into the periorbital area (22 cases). Other ancillary treatments are listed in Table 4. Among the concomitant surgeries, 5 and 9 patients underwent concurrent and subsequent buccal fat pad excision within 12 months after the modified TCLB, respectively, and it was the most frequent surgery performed with the modified TCLB. Other concomitant surgeries are shown in Table 5. The number of revision surgeries at other clinics (20 cases, 6.7%) was greater than that of our clinic (17 cases, 5.7%), and 4 subsequent revision surgeries were performed within 12 months postoperatively. The revised cases for the modified TCLB are listed in Supplemental Table 3. The patient satisfaction rate of surgical results was recorded 12 months postoperatively by a blind evaluation. The scale ranged from 0 to 3 (0 = poor, 1 = no difference, 2 = better, 3 = much better). The results were 0 for 0%, 1 for 5%, 2 for 20%, and 3 for 75%.

**Table 4.** Ancillary Treatment

	Previously	Simultaneously	Subsequently	Total
Hyaluronic acid injection into the tear trough or nasojugal fold	16^a^	3	22	41
Botox injection in the periorbital regions	16^a^	2	4	22
HIFU at the face	13^a^			13
PRP injection in the lower eyelids	9^a^			9
Fat-dissolving injection into the face	2^a^			2
Local steroid injection in the lower eyelids			1	1

HIFU, high-intensity focused ultrasound; PRP, platelet-rich plasma. ^a^The number of treatments performed at other clinics.

**Table 5.** Concurrent Surgery

Surgery	Previously	Simultaneously	Subsequently	Total
Upper blepharoplasty	15 (1^a^)	3^a^	4^a^	22
Double eyelid stitching	21 (1^a^)			21
Buccal fat pad excision		5^a^	9^a^	14
Thread lift	9	1^a^		10
Facelift	4	2^a^	2^a^	8
Rhinoplasty	6			6
Transcutaneous lower blepharoplasty	4			4
Facial liposuction	3			3
Facial fat transfer	3			3
Facial osteotomy	3			3
Medial epicanthoplasty		2^a^		2
Body liposuction	2			2
Breast augmentation	2			2

^a^The number of surgeries performed at the Ginza Cuvo Clinic, Tokyo, Japan.

**Figure 5.**
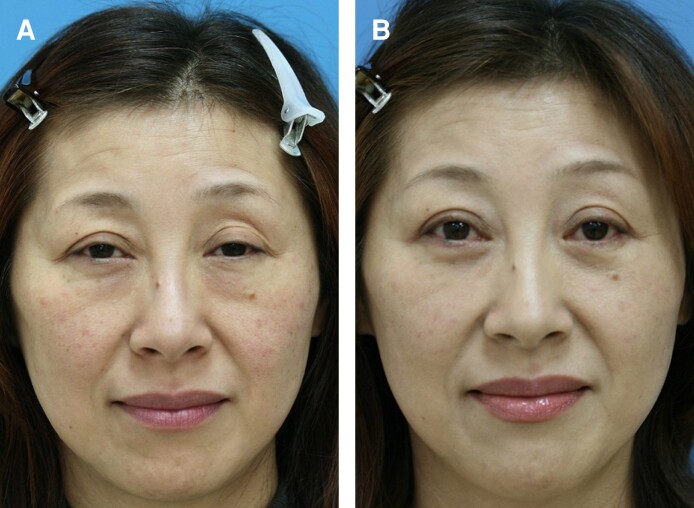
A 51-year-old female patient visited our clinic seeking to resolve difficulty in opening her eyes, a problem that had developed gradually over the last few decades. An ophthalmologist diagnosed the patient with blepharoptosis (more intense on the left side). The patient was also concerned about the dark circles and eyebags on the lower lid and came to the author's clinic for a second opinion. As observed in the preoperative photograph (A), she had enophthamia (sunken globe into the socket) and a ptotic upper lid with mild lower lid symptoms. The patient was advised to undergo a modified transconjunctival lower blepharoplasty for lower lid symptoms, expecting some resolution of the upper lid symptoms. A 12-month postoperative photograph (B) shows improvement in both the upper and lower lid symptoms. In particular, her ptotic upper lids improved enough to gain an eye-opening effect without any intervention to the upper lids. As a result, subsequent upper lid surgery has been postponed for the time being. Preoperative palpebral fissure heights for the right and left sides were improved from 6.3 mm and 6.0 mm to 9.5 mm and 9.0 mm postoperatively. The last photograph was taken on June 5, 2023.

**Figure 6.**
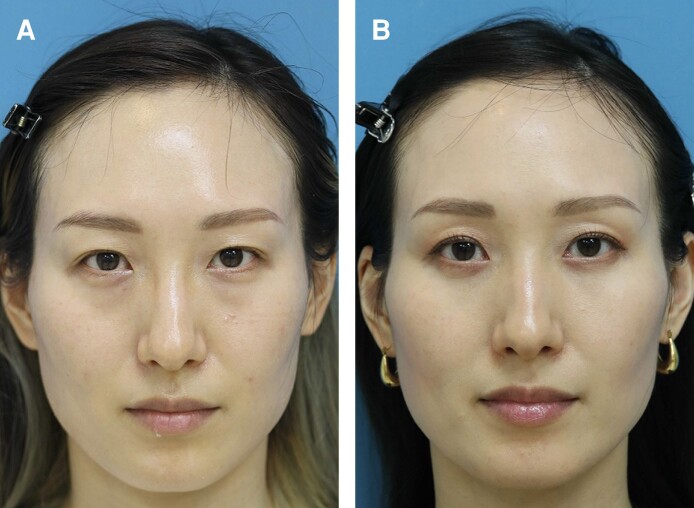
A 34-year-old female presented to our clinic with baggy lower lids and minor dark circles (A). She underwent a modified transconjunctival lower blepharoplasty for these symptoms. The 12-month postoperative photograph shows improvement of the lower lid symptoms, and the eye-opening effect was also obtained that she did not anticipate (B). Preoperative palpebral fissure heights for the right and left sides were improved from 9.5 mm and 9.8 mm to 10.2 mm and 10.6 mm postoperatively. The last photograph was taken May 3, 2023.

**Figure 7.**
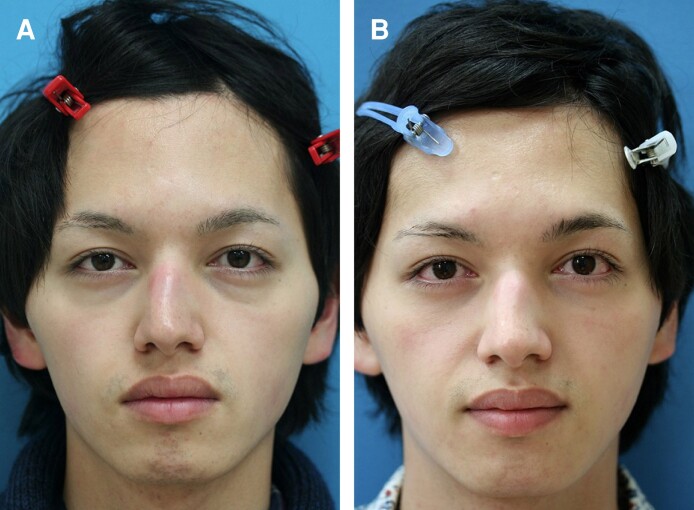
A 24-year-old male presented to our clinic wanting resolution of dark circles and tear trough deformity (A). He met several dermatologists and asked for opinions on how to solve them. He then underwent skincare treatments, such as bleaching skin care and laser therapy, but none were effective. He then decided to resort to surgery. He underwent a modified transconjunctival lower blepharoplasty, and the lower lid symptoms were significantly improved with a noticeable eye-opening effect (B). Preoperative palpebral fissure heights for the right and left sides were improved from 9.6 mm and 9.8 mm to 10.4 mm and 10.7 mm postoperatively. The last photograph was obtained on January 24, 2023.

**Figure 8.**
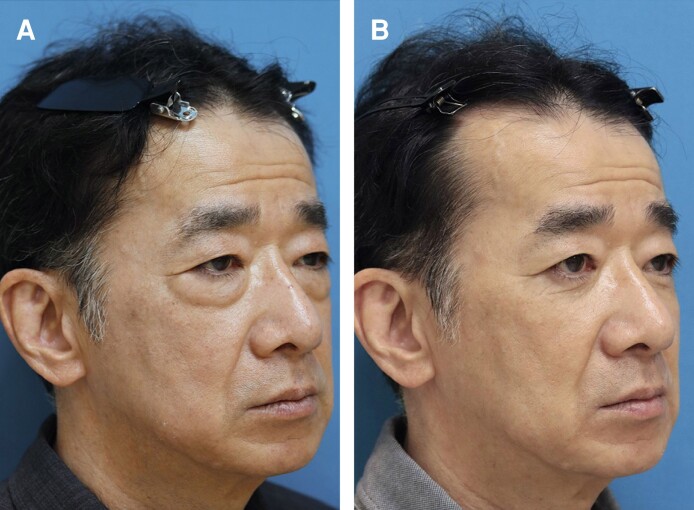
A 60-year-old male presented to our clinic wanting to improve his large eyebags, which bulged out to the lateral end of the lower lid (A). He underwent a modified transconjunctival lower blepharoplasty and his remarkable eyebags were significantly improved after the surgery, accompanied by the eye-opening effect as seen in the 12-month postoperative photograph (B). Preoperative palpebral fissure heights for the right and left were improved from 6.1 and 6.3 mm to 8.9 and 8.5 mm postoperatively. The final photograph was taken on October 7, 2023.

None of the patients had any serious complaints. Postinflammatory pigmentation in the lower eyelids occurred in 17 patients (5.7%) and was the most frequent complication. Tear trough deformity was exaggerated in 13 patients (4.3%). Postoperative hollowness and wrinkles at the lower lid appeared in 9 (3%) and 5 patients (1.6%), respectively. Most of these complications were temporary and resolved within 3 to 6 months postoperatively. In cases of prolonged recovery from these complications, hyaluronic acid was injected into the affected areas to expedite recovery. Chemosis was also seen in 4 patients (1.3%), and 3 of those 4 patients had previous lower blepharoplasty by either the transcutaneous or transconjunctival approach. The chemosis resolved within 3 to 4 weeks with steroid eye drops. Three patients (1%) had ecchymosis of the unilateral lower lid, although no bleeding tendency was observed in these patients in the preoperative hematological test. Ecchymosis resolved naturally within 1 to 2 weeks postoperatively. Injury to the zygomaticofacial nerve, a branch of the maxillary nerve (trigeminal V2), occurred in 2 patients (0.6%). There was minor sensory loss (paresthesia) in the affected region, which resolved naturally within 3 to 6 months postoperatively in 1 case. However, 1 male patient had prolonged sensory loss for more than 6 months, accompanied by a subsequent tingling sensation. The patient was referred to a neurologist for further examination; however, there were no specific findings. The patient was treated with oral vitamin B12, and the paresthesia diminished within 2 years postoperatively. Unilateral ectropion occurred in 2 senile female patients (0.6%). The affected lower eyelid was fixed with a Steri-Strip for 2 to 3 weeks postoperatively and resolved without further intervention. No cases of infection were reported (0%). All possible complications and the rate of occurrence in this study are presented in Supplemental Table 4. Typical cases of the eye-opening effect obtained with the modified TCLB are shown in Figures 5 and 6 and Supplemental Figures 1-3 (female cases) and Figures 7 and 8 (male cases).

## DISCUSSION

Typically, the initial signs of facial aging are evident in the upper eyelids. For instance, drooping due to attenuation of the aponeurosis of the levator palpebrae muscle, which plays a critical role in elevating the upper eyelids, is diagnosed as blepharoptosis.^18,19^ Blepharoptosis is characterized by an abnormal low-lying upper eyelid margin with the eye in primary gaze.^20^ Surgical intervention is sought in severe cases with favorable results being obtained when well-established protocols are used.^21^ Blepharoptosis is broadly classified into 2 types: congenital and acquired. Although the former is uncommon, it should be treated at an early stage to prevent the onset of amblyopia.^22^ The latter is usually observed in aging populations, and most cases of acquired blepharoptosis are of the aponeurotic (involutional) type, resulting from stretching, dehiscence, or disinsertion of the levator aponeurosis.^23^ The aponeurotic type is often exacerbated by mechanical friction. It is occasionally observed in middle-aged Asian women whose aponeurosis is thinner and who wear hard contact lenses for a long time.^24^ Correction of aponeurotic blepharoptosis involves shortening or plication of the aponeurosis.^23^ Dermatochalasis, or pseudoblepharoptosis, is a skin laxity of the upper eyelids that mimics blepharoptosis, requiring meticulous diagnosis due to the different treatment strategies. Signs of early aging also occur in the lower lids, represented by eyebags, likely to occur in patients with relatively large amounts of LOFC when the supporting tissues become attenuated.^15^ Surgical protocols and their efficacies for lower lid symptoms have been well established, and there are 2 approaches to lower blepharoplasty: transcutaneous or transconjunctival.^25,26^ A subciliary incision during the transcutaneous approach invades the pretarsal orbicularis oculi muscle, in which capillaries and peripheral nerves play an important functional role. Thus, the incision should be made carefully to preserve anatomical structures. The risk of ectropion, the eversion of the lower lid, must always be considered with the transcutaneous approach, especially in patients with a loose canthus.^1,27^ The result may not be aesthetically pleasing and may lead to functional problems such as dryness or discomfort. Therefore, the risk factors for ectropion should be scrutinized preoperatively. Concurrent canthopexy or canthoplasty should be considered as a preventive measure against ectropion. The greatest advantage of the transconjunctival approach is the preservation of the anatomical structures of the lower eyelids. However, if mere fat evulsion in TCLB is performed in elderly patients with lax skin, the effect may worsen due to contour deformity. Evidence of inappropriate TCLB can be seen through the presence of dents, hollowness, or deep wrinkles on the lower lid. Therefore, there has been a reduction in applications for traditional TCLB and it is now typically only performed in carefully selected cases in younger patients. Instead, the trend in TCLB has been LOFC preservation rather than extirpation.

Recently, refined techniques for tear trough deformities during TCLB have progressed significantly. These innovative techniques include LOFC repositioning and orbital unipedicled fat pad repositioning.^28-30^ This demonstrated excellent results, compensating for the insufficiencies of traditional TCLB methods.^31^ Evidence of well-managed tear trough deformity with TCLB has also been demonstrated by autologous fat tissue transfer, harvested from the trunk or thigh, or by using minced fat pellets created from the LOFC itself.^32,33^ However, the former technique lacks accuracy due to the volume of transferred fat absorbed.^34^ The latter technique is more accurate due to a lower absorption rate. Both techniques were compared in a recent study adopting a minced free fat graft harvested from the LOFC with the rotation flap method using a pedicled nasal LOFC. Both techniques yielded excellent results with little difference between the methods.^35^ However, the risks associated with autologous fat transfer techniques must be considered. Contour irregularities around the lower lids due to fat granulomas and overgrafting are often observed.^36^ Intravascular injection during periorbital fat transfer should be avoided due to risks of blindness and cerebral infarction.^37,38^

Although applications for upper and lower lid surgeries are differentiated depending on patient presentation, it is important to consider how each lid symptom might correlate and how one surgery might affect the other. However, to the best of our knowledge, there have been only a few scientific papers concerning the mutual influence of each lid symptom. The globe is fully mobile, as if it is a gyroscope floating inside the orbit. If the upper lid is slightly pressed, this tiny force is immediately transmitted toward the lower lid and prolapses the LOFC anteriorly. Conversely, if the lower lid is pressed horizontally by a slight external force, the upper lid bulges anteriorly. Given that both lids intimately affect each other, there is a possibility that even a slight dynamic force inflicted by periorbital surgery might affect both lids. For example, exophthalmos is a protrusion of the globe. Although exophthalmos is associated with retrobulbar fat expansion, some improvement in proptosis has been achieved by removing the LOFC during lower blepharoplasty.^39^ Evidence suggests that this improvement is brought about by LOFC excision, yielding more space for the globe to recede in the orbit.

However, enophthalmia is related to extra orbital fat prolapse and is an involutional change in the relationship between the globe and socket, causing abnormally sunken eyeballs. A previous study stated that the weight burden of the LOFC drags the globe down due to the aging laxity of suspended tissues at the lower lid, including the lateral canthus tendon and the LL inserted into the capsulopalpebral fascia and inferior rectus muscle.^14^ The pathophysiology of enophthalmia is based on knowledge from previous studies and the author's experience of surgeries in which the distal part of the central and lateral LOFC gradually slides anteriorly with aging, impinging into a narrow space between the AELL, LOTA, and LL over the inferior orbital rim. The herniated LOFC gradually becomes larger as the blood and lymph fluid circulations of the herniated LOFC are impeded by the bottlenecked exit surrounded by ligaments. This vicious circle of LOFC impingement may increase the internal pressure in the orbital socket, causing the globe to recede. As evidence of this pathophysiology, when the AELL and ORL are relaxed, the lateral LOFC pops out, demonstrating the considerable pressure exerted by these ligaments. Dissection and severing of the AELL, the TLPORL, and part of the lateral retinaculum contributed to solving the bottleneck exit of the lateral LOFC. This allowed the pressure to release inside the orbital socket and may assist in resolving issues of the sunken globe to increase the eye-opening effect relative to the forward movement of the globe, as shown in Supplemental Figure 4. For instance, dermatochalasis may occur because the globe is relatively covered by the upper lid as it recedes into the socket. It is likely that Asian eyes are smaller than Occidental eyes, and this feature might predispose them to enophthalmia. The amount of LOFC was not necessarily related to symptom development, as shown in Figure 5A,B, which depicts a relatively small amount.

It was impossible to examine eye protrusion values using 3D photographs because analysis in the front-back direction was not sufficiently accurate to evaluate proptosis. The Hertel exophthalmometer was recently introduced to determine preoperative and postoperative proptosis values. Preliminary data of preoperative proptosis values in 32 new patients (aged 45.4 [12.6] years; range, 24-78 years) who underwent the modified TCLB in 2023 were 13.2 [2.3] mm (range, 8-18 mm) for the right eye and 13.3 [2.2]mm (range, 8-18 mm) for the left eye. These patients were found to have globes that were more sunken compared with average proptosis values for Asians (14.4 mm). Notably, the eye protrusion value of a typical patient with enophthalmia (Figure 5A,B) was small (9 mm on both sides). The 12-month postoperative eye protrusion values in the new patient group are now being collected, with the expectation that data will show postoperative improvement of sunken globes. The basis of the modified TCLB is the decompression of the LOFC by deframing supporting tissues. First, the LOFC was detached anteriorly from the preseptal orbicularis oculi until the dissection reached the inferior orbital rim. Here the ORL and tear trough ligament running over the arcus marginalis were released simultaneously. Next, the AELL running between the central and lateral LOFCs was identified and severed. With transection of the AELL, the central and lateral LOFCs become freely mobile, as documented in a previous paper.^1^ The TLPORL was also widely dissected to obtain sufficient space for the lateral LOFC to settle inside the posterolateral orbital socket. Part of AELL runs toward the medial retinaculum over the nasal LOFC, and this was also transected for deframing purposes. Also identified and partially transected was the lateral retinaculum at the posterolateral orbital socket. Finally, the excess portion of each LOFC was trimmed or transpositioned as necessary.

The main concern of TCLB is overresection of the LOFC, resulting in a hollowed lower lid. It often occurs in the original TCLB method where LOFCs are forcibly pushed anteriorly with the aging attenuation of supporting tissues. This excess of LOFC is likely to be resected with conventional TCLB, causing a hollowed lower lid due to overresection. Even worse, LOFC resection cannot be distributed evenly by old-style TCLB because the frame (ligaments and septum) holds the LOFC intact, so that resection can cause indentation in the lower lid. The modified approach to TCLB enables minimal resection of the true excess part of the LOFC with the distal part receding into the original position after the deframing and decompression maneuver. Therefore, this process is necessary to prevent overresection of the LOFC. Negative impacts of LOFC resection can be easily avoided using these maneuvers with evenly distributed mobilized LOFCs as shown in Supplemental Figure 5. Therefore, the positive results obtained using the modified TCLB highlight that prioritizing deframing and decompression of the LOFCs during TCLB leads to more favorable results.

In the case of a larger LOFC, resection yields more space and better positioning of the globe in the orbit. Removing the weight burden of excess LOFCs may also contribute to the elevation of the midface. In contrast, preservation of adequate amounts of LOFC is necessary for midface fullness for those with deflated or lean faces as overresection would increase the hollowed appearance. Interestingly, the resected lateral LOFC was greater than the nasal and central LOFCs (Supplemental Table 1). This demonstrates that resection of the lateral LOFC offered the greatest improvement to lower lid symptoms, despite the possibility of pronounced symptoms in the nasal or central LOFC regions such as eyebags or tear trough deformities. However, resected fat from the lateral LOFC may be mistakenly measured as greater than the actual amount if any fat pad from the central LOFC is included. If both central and lateral LOFCs are treated as a single unit after transection of the AELL, the central LOFC might be resected as part of the lateral LOFC as the central LOFC is dragged down laterally for the resections. In contrast, the protrusion vector of the nasal LOFC is in the horizontal direction, and the bulging part should be adequately resected.^40^

The concomitant surgeries are presented in Table 5. Buccal fat pad excision for slimming or lifting the face was most frequently performed for total facial rejuvenation due to its minimal invasiveness in patient groups such as these. The conversion rate for upper lid surgery after the follow-up period was low. Patients with modified TCLB showed concurrent improvement in the upper lids with a typically favorable satisfaction rate. Although progress during the first couple of postoperative months was not obvious in most cases, it gradually improved 6 to 12 months postoperatively. Minor complications were temporary and diminished naturally in most patients (Supplemental Table 4). Patient concerns were postoperative dents, hollowness, and wrinkles at the lower lids. However, the actual occurrence of these complications was much lower than expected. In such occurrences, minor touch-up treatments with injectable fillers (hyaluronic acid) succeeded in accelerating recovery. Although injury to the peripheral sensory nerve (zygomaticofacial nerve) did occur unexpectedly, the sensory loss was reversible and resolved postoperatively. It is believed that the cause of nerve injury was excessive mechanical force exerted by a retractor device, pulling the lower lid anterolateral over a prolonged time.

In conclusion, dermatochalasis (pseudoblepharoptosis) involving enophthalmia is partially caused by anatomical defects of the lower lid due to involution. If upper lid symptoms are within a mild or physiological range accompanied by typical lower lid symptoms, a modified TCLB method should be considered. This not only resolves lower lid symptoms, but also offers simultaneous improvement of upper lid symptoms. If further improvement to the upper eyelids is required, subsequent upper lid surgery can be considered. This surgical protocol can yield better results at the conclusion of the total periorbital rejuvenation as the burden on the upper lids may be resolved without additional surgery. The pathophysiology of the eye-opening effect with modified TCLB is a hypothesis inspired by a previous study on enophthalmia, and it is not currently possible to reach reliable conclusions as surgical outcomes vary among individuals.^14^ Modified TCLB is principally performed for lower lid symptoms, and not all patients undergoing this surgery gain an improved effect on the upper eyelids. Another limitation of this study was an insufficient number of patients to reach a definitive conclusion regarding the eye-opening effect of the modified TCLB. Therefore, this study should be carried out with a larger sample size and longer-term follow-up.

Traditionally, lower blepharoplasty has only been performed to improve lower eyelid symptoms, and its effects have been limited to the lower eyelid. However, by modifying and extending the treatment technique, it has been proven for the first time that the positive effects can extend not only to the lower eyelid, but also improve the symptoms of the entire orbit, such as eyelid ptosis and sunken eyeballs in the socket (enophthalmia). This finding may contribute to the field of oculoplastic surgery as a new treatment strategy that prioritizes lower eyelid treatment in patients with concurrent upper lid ptosis. Finally, matching the globe and socket proves indispensable for a healthy and aesthetically pleasing eyelid appearance. Therefore, restructuring of the lower orbit with lower blepharoplasty should be reaffirmed as a good strategy for total beautification and rejuvenation of the periocular region. Anatomical alterations in the orbit ensuring this phenomenon with this surgery should be further investigated by other means, such as comparison of 3D pre- and postoperative MRI.

## CONCLUSIONS

A modified TCLB performed for lower lid symptoms also offers improved results to the upper lid. These effects can result from the deframing and decompression of the LOFCs to acquire anatomical relevance in the orbit with sufficient resection of the lateral LOFC, creating more space for the globe to settle back into the desired position. This results in corrected enophthalmia and associated ptotic upper lid. Therefore, priority should be given to performing the modified TCLB in patients seeking resolution of symptoms in both lower and concurrent minor upper lids.

## Supplemental Material

This article contains supplemental material available online at https://doi.org/10.1093/asj/sjae205.

## Supplementary Material

sjae205_Supplementary_Data
