# Supplementary material for: Eye-Opening Effect Achieved by Modified Transconjunctival Lower Blepharoplasty
Source: Aesthet Surg J. 2024 Oct 17;45(2):126–35. doi: 10.1093/asj/sjae205 (PMC11852279; doi:10.1093/asj/sjae205)
Supplement: sjae205_Supplementary_Data [file sjae205_supplementary_data.zip › Supplemental Table 1 ( Excised fat weight).docx]

|  | Nasal | Central | Lateral | Total |
| --- | --- | --- | --- | --- |
| Right | 0.12±0.09 (0-0.4) | 0.1±0.06 (0-0.3) | 0.21±0.14 (0-0.6) | 0.43±0.24 (0-1.2) |
| Left | 0.14±0.1 (0-0.6) | 0.11±0.07 (0-0.4) | 0.17±0.13 (0-0.7) | 0.42±0.25 (0-1.5) |
