# Supplementary material for: Eye-Opening Effect Achieved by Modified Transconjunctival Lower Blepharoplasty
Source: Aesthet Surg J. 2024 Oct 17;45(2):126–35. doi: 10.1093/asj/sjae205 (PMC11852279; doi:10.1093/asj/sjae205)
Supplement: sjae205_Supplementary_Data [file sjae205_supplementary_data.zip › Supplemental Table 2 (Vertical widths between upper and lower palpebral fissures ).docx]

|  | Preoperatively | 12 months postoperatively | P-value |
| --- | --- | --- | --- |
| Right | 8.41±1.15 (6.1-10.7) | 9.26±0.95 (6.4-11.1) | P<0.01 |
| Left | 8.41±1.12 (5.5-10.4) | 9.21±0.94 (6.2-11.1) | P<0.01 |
