# Supplementary material for: Eye-Opening Effect Achieved by Modified Transconjunctival Lower Blepharoplasty
Source: Aesthet Surg J. 2024 Oct 17;45(2):126–35. doi: 10.1093/asj/sjae205 (PMC11852279; doi:10.1093/asj/sjae205)
Supplement: sjae205_Supplementary_Data [file sjae205_supplementary_data.zip › Supplemental Table 3 (Number of Revision surgery).docx]

|  | **Number of Revision Surgeries** | **Total** |
| --- | --- | --- |
| **Our clinic** | 3 (left)  1 (right)  13 (both sides) | 17 |
| **Other clinics** | 20 (both sides) | 20 |
