# Supplementary material for: Eye-Opening Effect Achieved by Modified Transconjunctival Lower Blepharoplasty
Source: Aesthet Surg J. 2024 Oct 17;45(2):126–35. doi: 10.1093/asj/sjae205 (PMC11852279; doi:10.1093/asj/sjae205)
Supplement: sjae205_Supplementary_Data [file sjae205_supplementary_data.zip › Supplemental Table 4 (Complications and Their Rates of Occurrence).docx]

| Complications | Rate of occurrence (%) |
| --- | --- |
| Pigmentation | 5.7 |
| Tear trough deformity | 4.3 |
| Hollowness | 3 |
| Wrinkles | 1.6 |
| Chemosis | 1.3 |
| Ecchymosis | 1 |
| Paresthesia | 0.6 |
| Ectropion | 0.6 |
| Infection | 0 |
