# Supplementary figures and images for: Eye-Opening Effect Achieved by Modified Transconjunctival Lower Blepharoplasty
Source: Aesthet Surg J. 2024 Oct 17;45(2):126–35. doi: 10.1093/asj/sjae205 (PMC11852279; doi:10.1093/asj/sjae205)

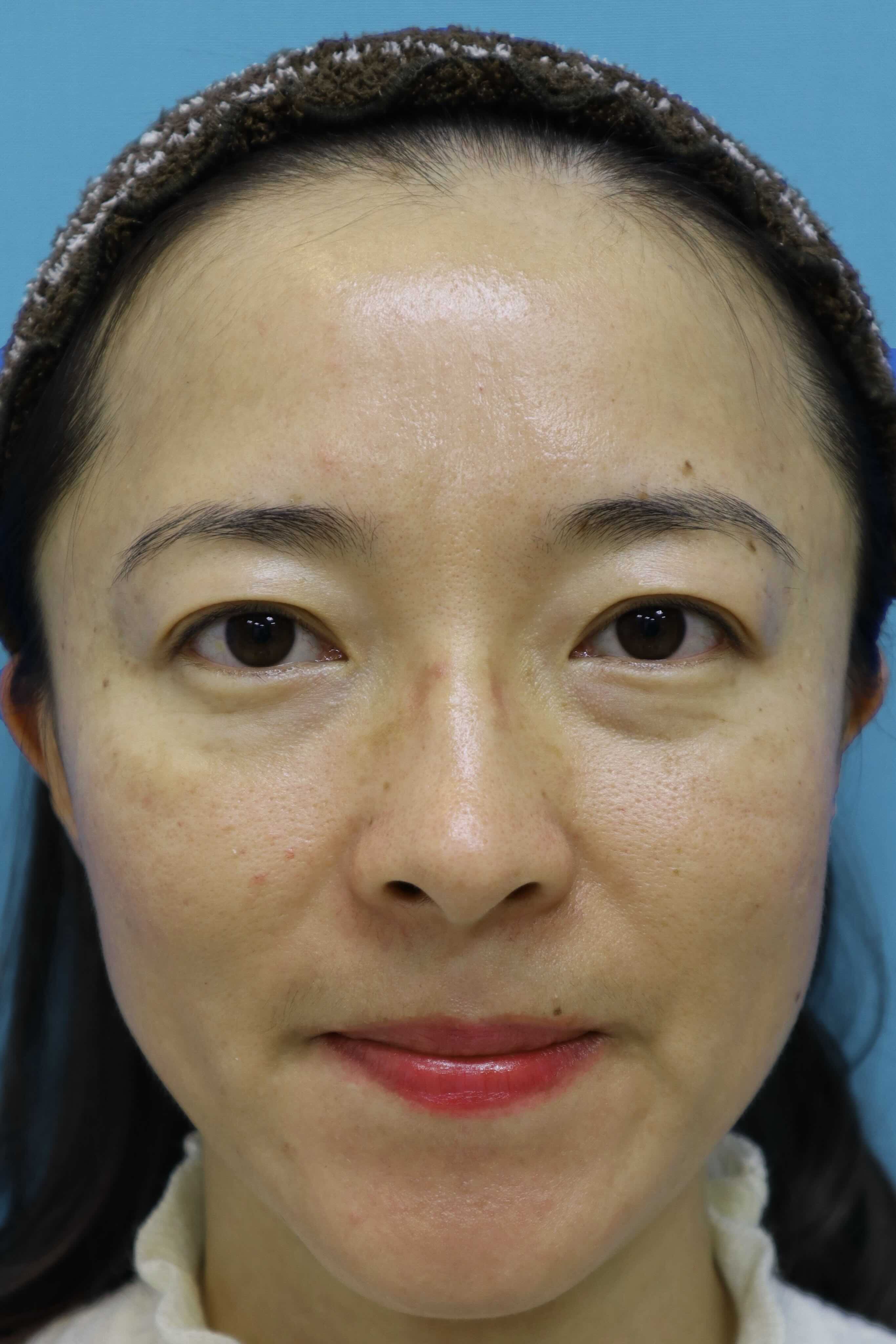

Supplement: sjae205_Supplementary_Data [file sjae205_supplementary_data.zip › SuppFig1a.jpg]

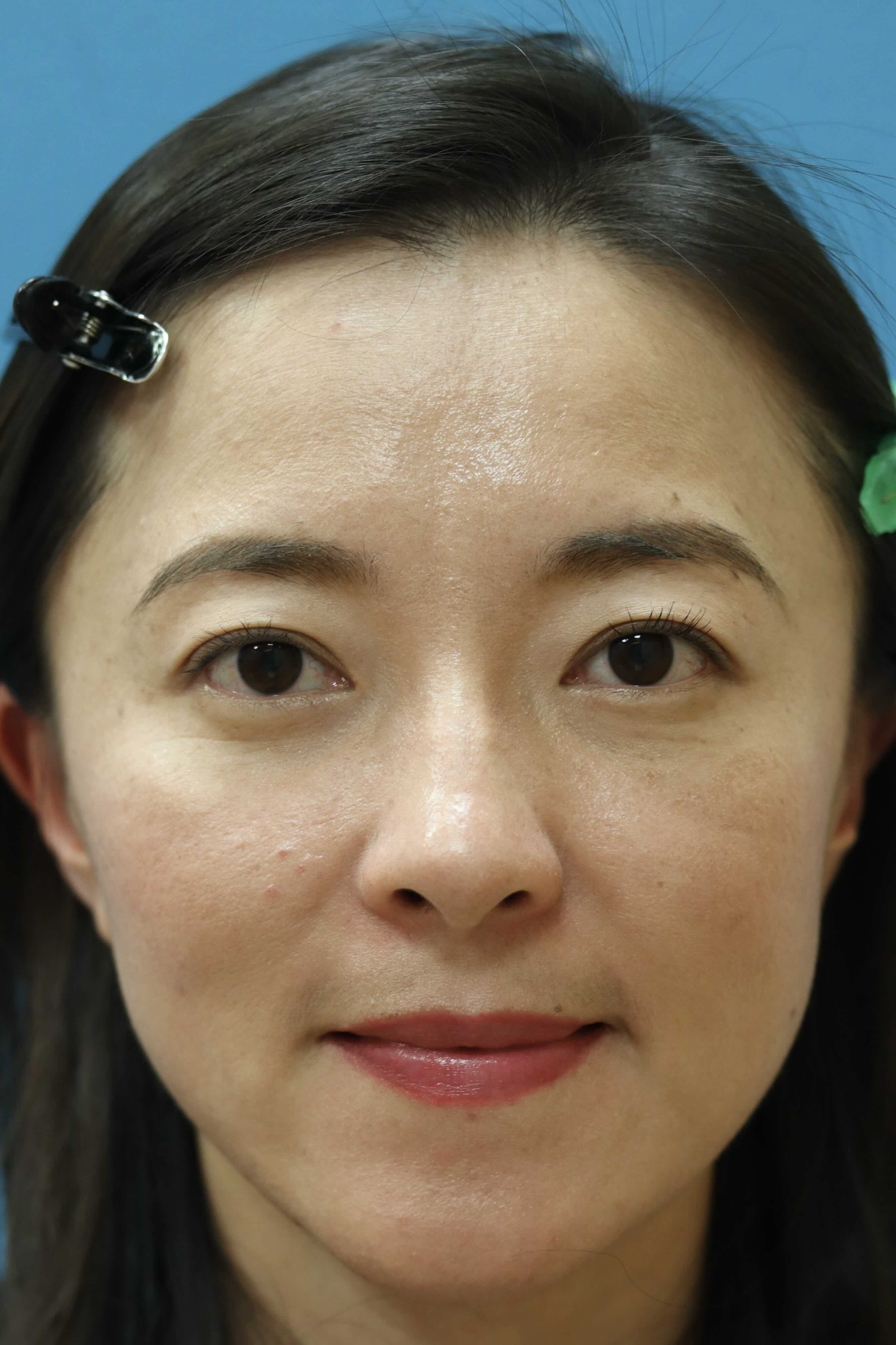

Supplement: sjae205_Supplementary_Data [file sjae205_supplementary_data.zip › SuppFig1b.jpg]

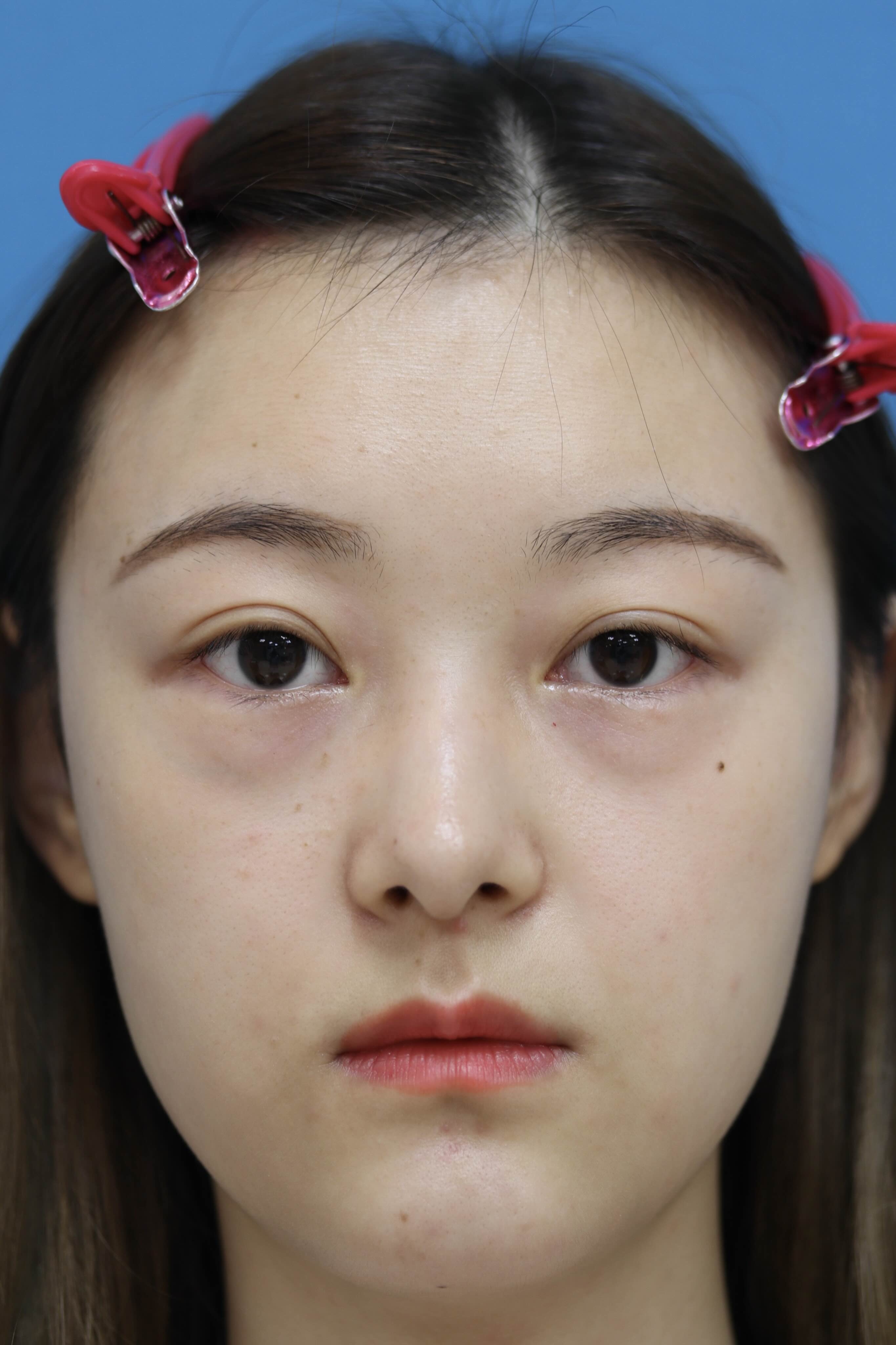

Supplement: sjae205_Supplementary_Data [file sjae205_supplementary_data.zip › SuppFig2a.jpg]

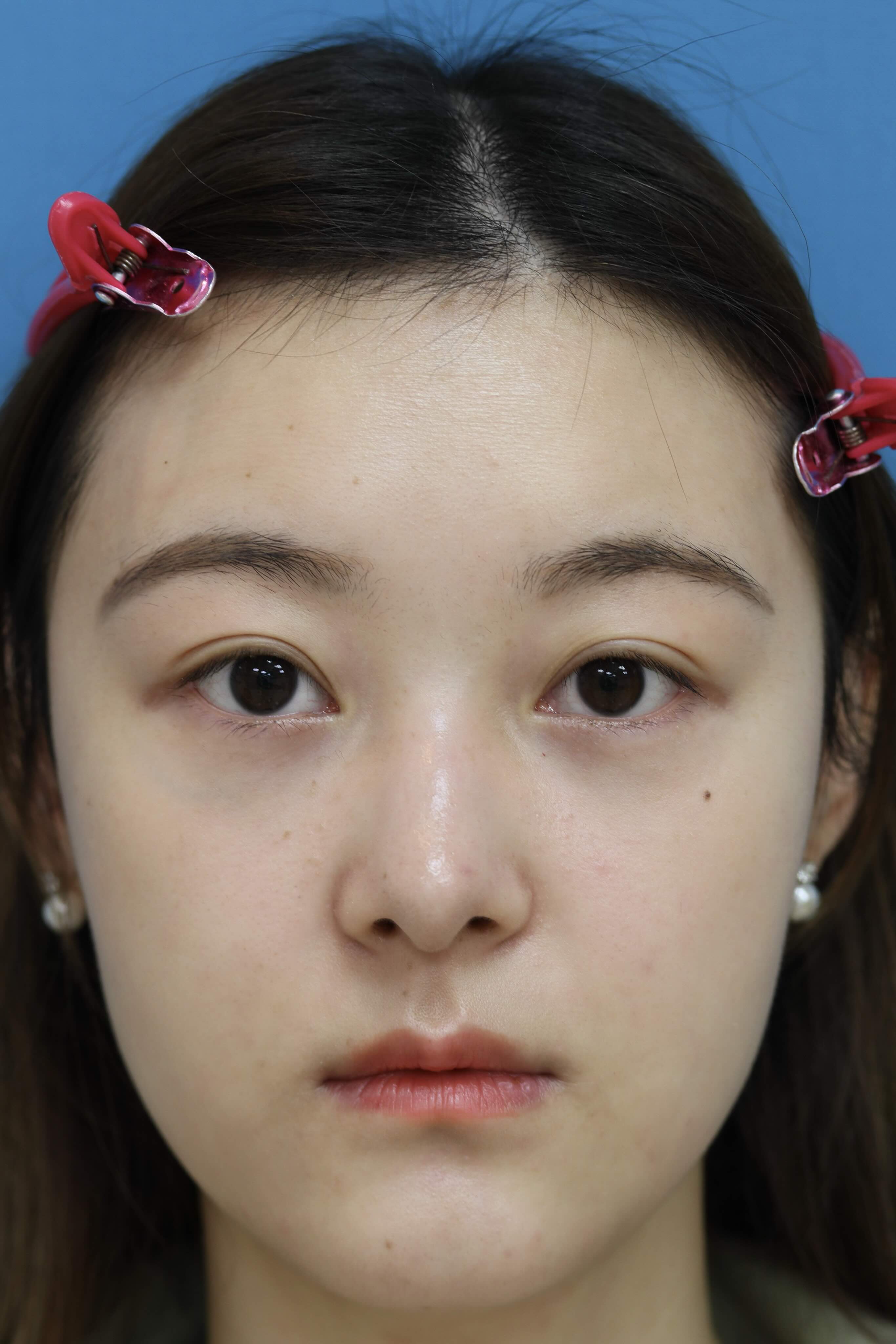

Supplement: sjae205_Supplementary_Data [file sjae205_supplementary_data.zip › SuppFig2b.jpg]

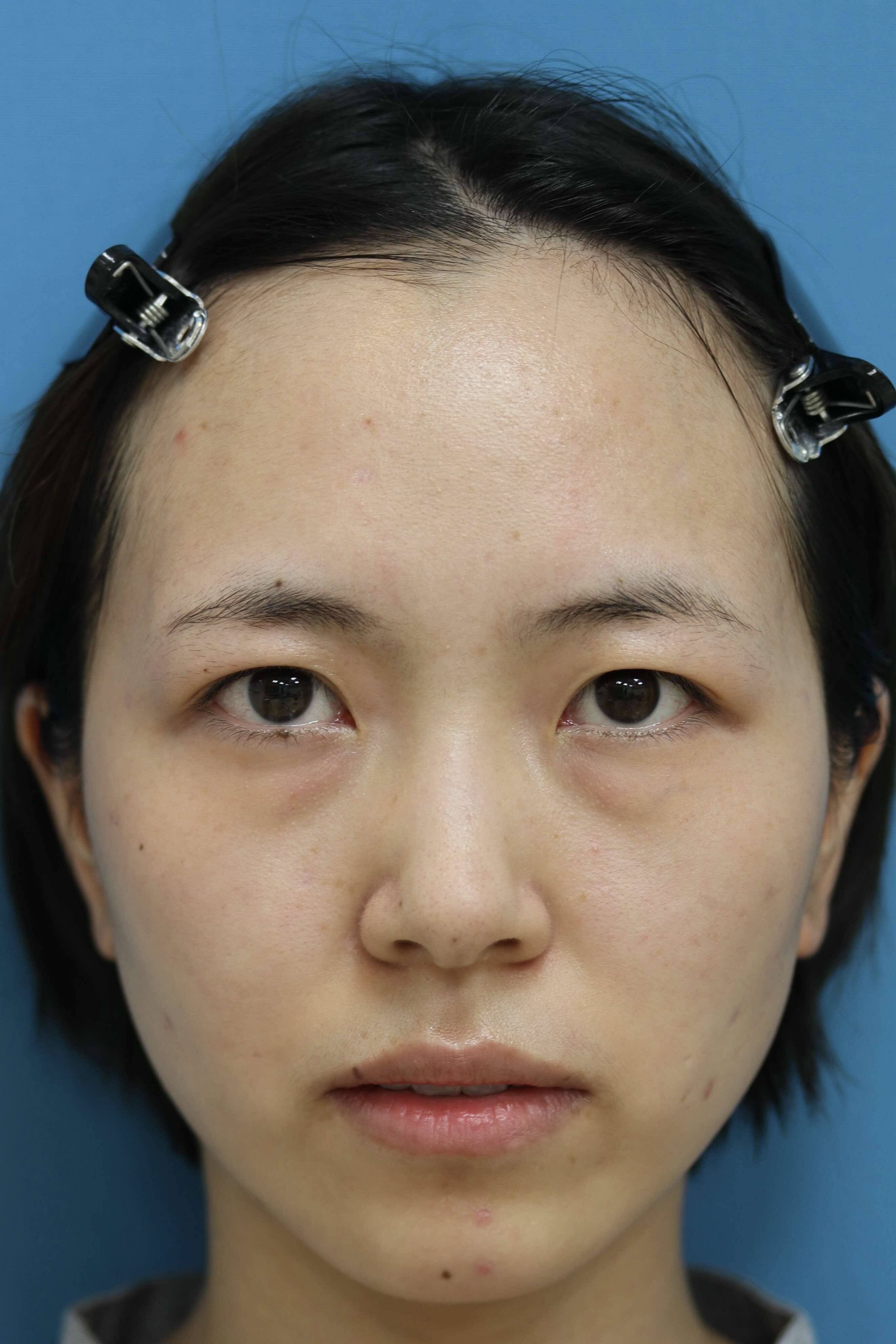

Supplement: sjae205_Supplementary_Data [file sjae205_supplementary_data.zip › SuppFig3a.jpg]

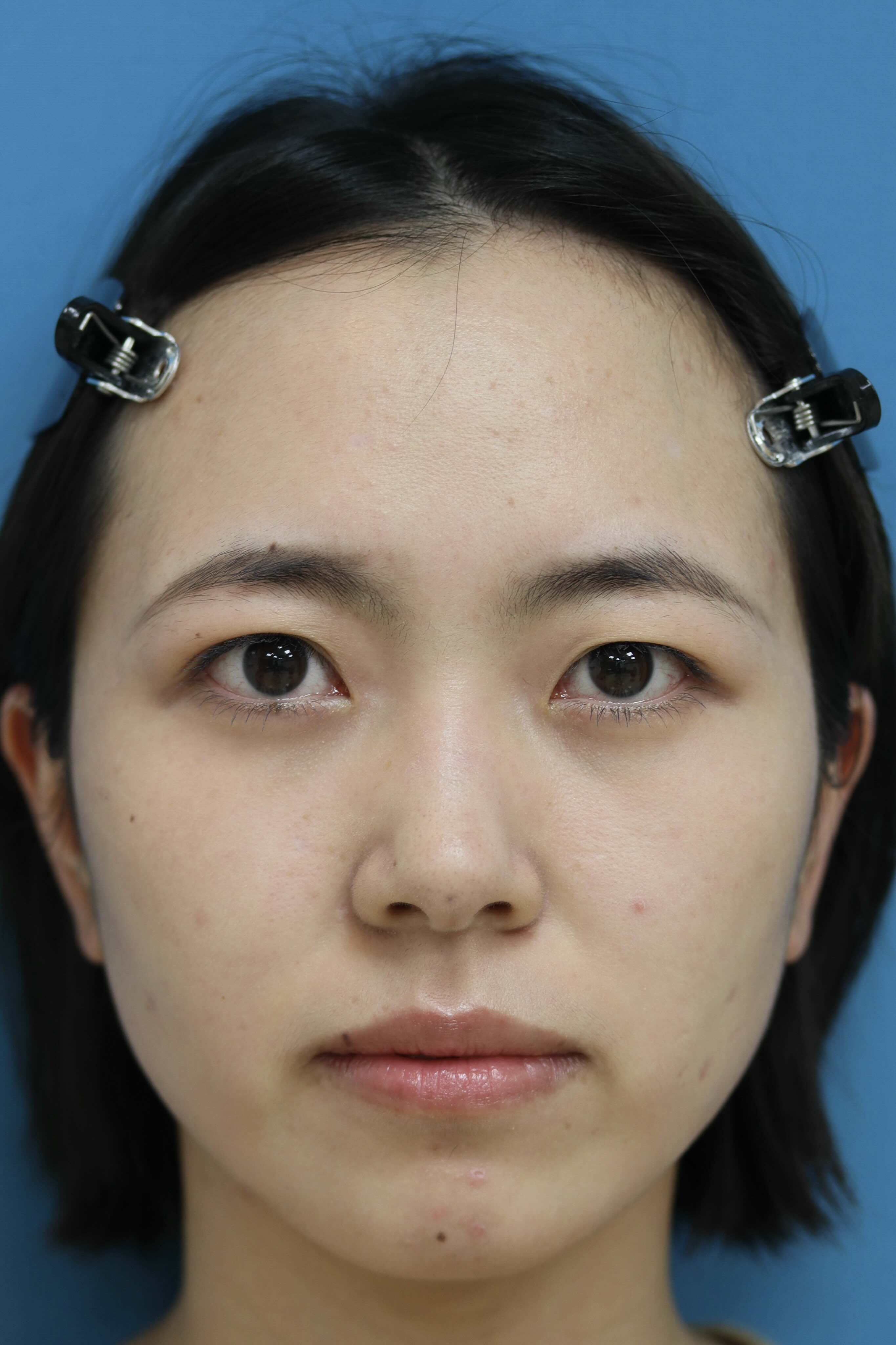

Supplement: sjae205_Supplementary_Data [file sjae205_supplementary_data.zip › SuppFig3b.jpg]

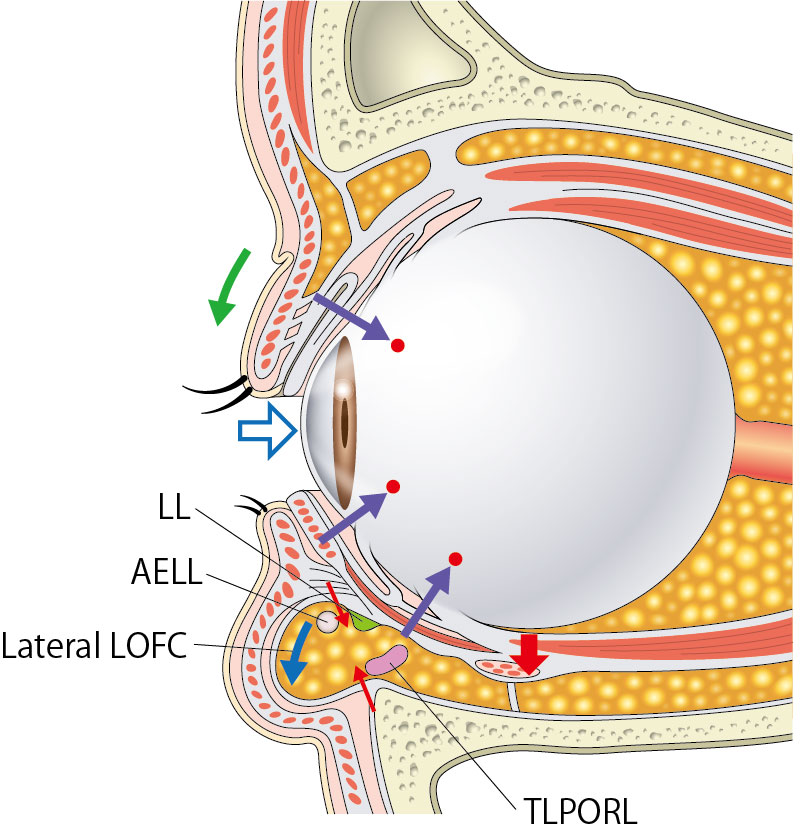

Supplement: sjae205_Supplementary_Data [file sjae205_supplementary_data.zip › SuppFig4a.jpg]

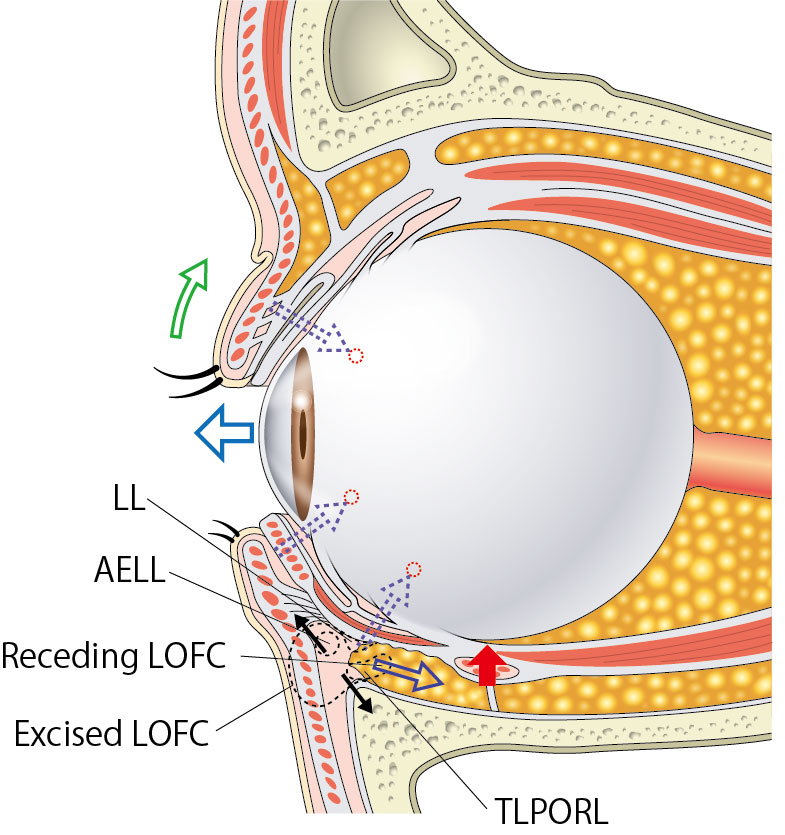

Supplement: sjae205_Supplementary_Data [file sjae205_supplementary_data.zip › SuppFig4b.jpg]

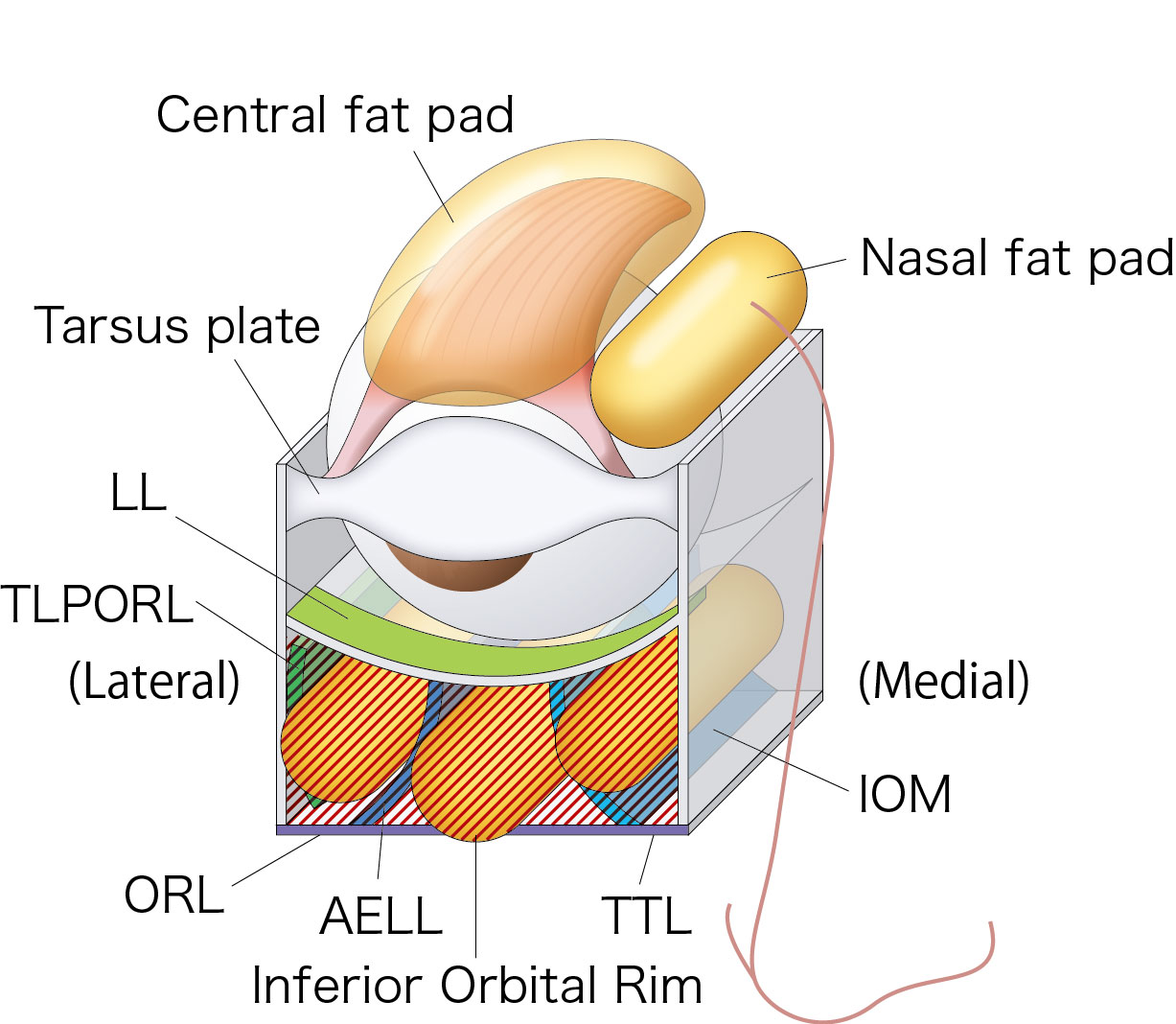

Supplement: sjae205_Supplementary_Data [file sjae205_supplementary_data.zip › SuppFig5a.jpg]

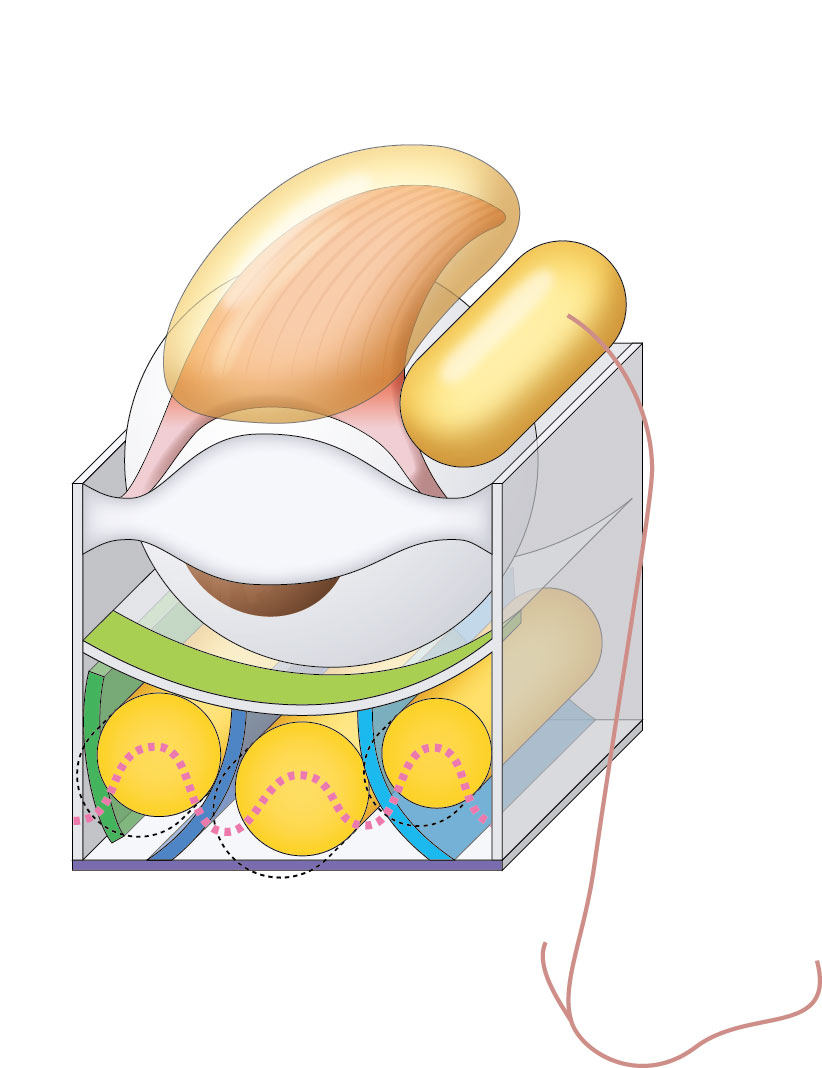

Supplement: sjae205_Supplementary_Data [file sjae205_supplementary_data.zip › SuppFig5b.jpg]

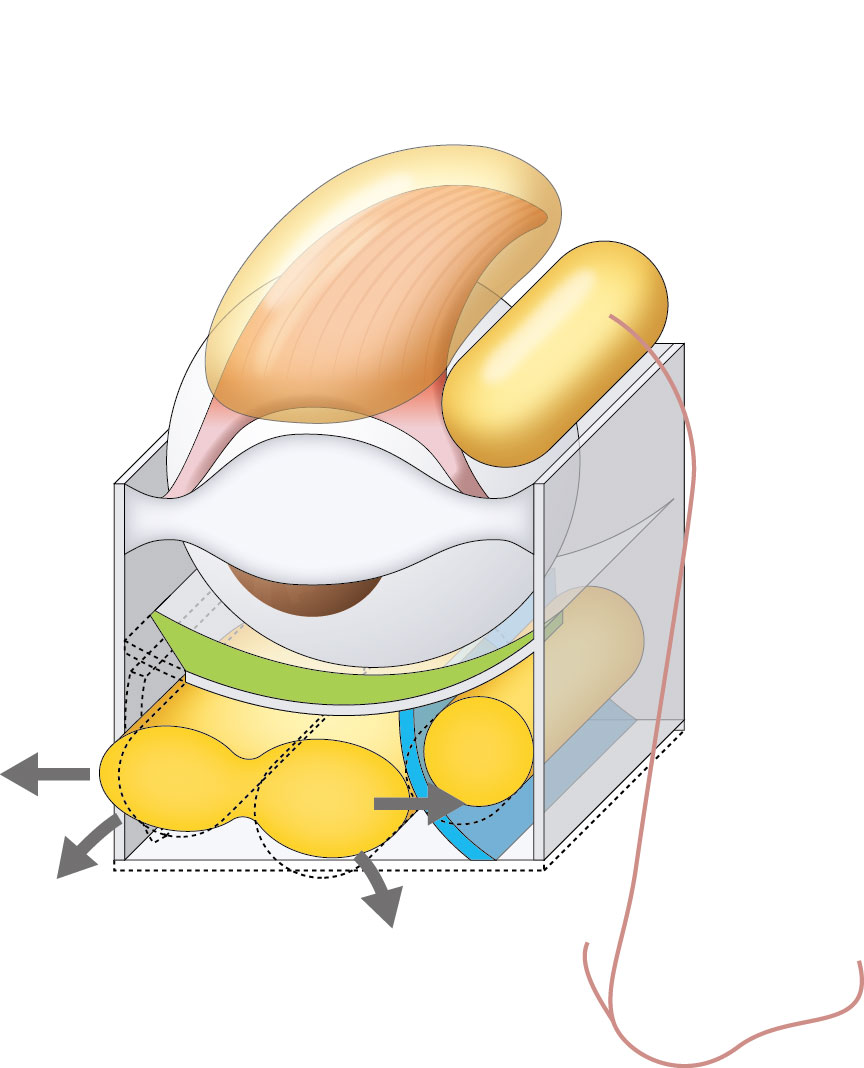

Supplement: sjae205_Supplementary_Data [file sjae205_supplementary_data.zip › SuppFig5c.jpg]
